# Application of Machine Learning in Intelligent Medical Image Diagnosis and Construction of Intelligent Service Process

**DOI:** 10.1155/2022/9152605

**Published:** 2022-12-28

**Authors:** Zhihong Gao, Lihua Lou, Meihao Wang, Zhen Sun, Xiaodong Chen, Xiang Zhang, Zhifang Pan, Haibin Hao, Yu Zhang, Shichao Quan, Shaobo Yin, Cai Lin, Xian Shen

**Affiliations:** ^1^Department of Big Data in Health Science, The First Affiliated Hospital of Wenzhou Medical University, Wenzhou, Zhejiang 325000, China; ^2^Department of Burn, Wound Repair and Regenerative Medicine Center, The First Affiliated Hospital of Wenzhou Medical University, Wenzhou 325000, China; ^3^Department of Radiology, The First Affiliated Hospital of Wenzhou Medical University, Wenzhou 325000, China; ^4^Department of Gastrointestinal Surgery, The First Affiliated Hospital of Wenzhou Medical University, Wenzhou 325000, China; ^5^Wenzhou Data Management and Development Group Co., Ltd., Wenzhou, Zhejiang 325000, China

## Abstract

The introduction of digital technology in the healthcare industry is marked by ongoing difficulties with implementation and use. Slow progress has been made in unifying different healthcare systems, and much of the globe still lacks a fully integrated healthcare system. As a result, it is critical and advantageous for healthcare providers to comprehend the fundamental ideas of AI in order to design and deliver their own AI-powered technology. AI is commonly defined as the capacity of machines to mimic human cognitive functions. It can tackle jobs with equivalent or superior performance to humans by combining computer science, algorithms, machine learning, and data science. The healthcare system is a dynamic and evolving environment, and medical experts are constantly confronted with new issues, shifting duties, and frequent interruptions. Because of this variation, illness diagnosis frequently becomes a secondary concern for healthcare professionals. Furthermore, clinical interpretation of medical information is a cognitively demanding endeavor. This applies not just to seasoned experts, but also to individuals with varying or limited skills, such as young assistant doctors. In this paper, we proposed the comparative analysis of various state-of-the-art methods of deep learning for medical imaging diagnosis and evaluated various important characteristics. The methodology is to evaluate various important factors such as interpretability, visualization, semantic data, and quantification of logical relationships in medical data. Furthermore, the glaucoma diagnosis system is discussed in detail via qualitative and quantitative approaches. Finally, the applications and future prospects were also discussed.

## 1. Introduction

Medical imaging plays an important role in clinical applications, life science research, etc. [1, 2]. Different modalities of medical imaging techniques generate discrete images through sampling or reconstruction, map values to the airspace, and form medical images that express the internal structure or function of an anatomical region [3–5]. From X-rays and ultrasound to computed tomography (CT), magnetic resonance imaging (MRI), and positron emission tomography (PECT), every innovation in imaging technology is an enrichment and observational capability for medical objects [6–9]. It has played a vital role in improving medical means and improving medical standards [10]. The development and progress of computer science have greatly improved the ability to interpret medical images, and deep learning [11] is one of the important research directions in machine learning. In recent years, deep learning has made remarkable achievements in the field of computer vision [12–14]. Significant progress has also been made in applying deep learning to lesion target segmentation, localization, detection, image registration, and fusion in medical images. Fast diagnosis, diagnosis time is greatly shortened [15].

Although medical diagnosis based on deep learning has made great progress [16–19], there are still some urgent problems to be solved in clinical practice .

**Figure 1:**
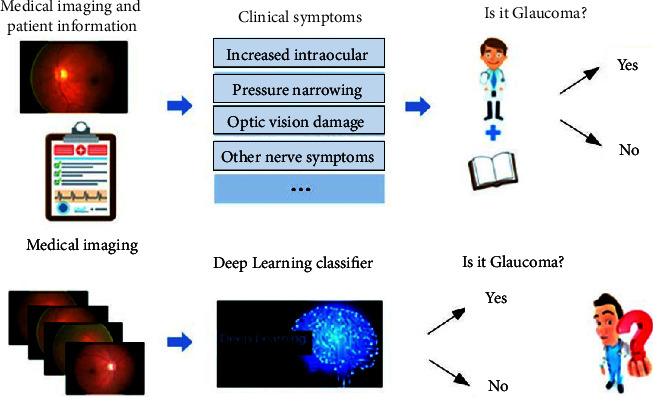
Comparison of diagnosis systems [3].

1. The generalization ability of data-driven deep learning algorithms is often questioned and challenged. Insufficient sample data and inconsistencies between the distribution of training samples and real samples will lead to a sharp drop in the performance of the algorithm. Different from natural image processing with powerful datasets, whether the model trained in the scenario of very few medical samples can be used for high-precision and sensitive medical image analysis is one of the points that has been questioned [20–23]. As reported by The Wall Street Journal on January 26, 2019, Google's deep learning algorithm for diagnosing diabetic retinopathy has been challenged in India's labs and hospitals due to poor imaging equipment in Indian hospitals. The developed algorithm cannot effectively identify low-quality images.
2. Adversarial examples raise deep concerns about the robustness of deep learning. Adversarial examples are examples that are slightly perturbed, which can cause the model to output incorrect results with high confidence. The emergence of this “ridiculous” phenomenon has forced people to explore deep learning methods to obtain robust output results.
3. Deep learning can automatically extract abstract features, and its prediction process is end-to-end. It only has direct results, cannot provide diagnostic basis, etiology or pathology, and cannot be fully trusted and accepted. For example, in the screening of glaucoma (see Figure 1), doctors can diagnose the disease through intraocular pressure detection, visual field detection, and manual inspection of the optic disc, combined with the patient's clinical symptoms and pathological reports, and give the cause and pathology. However, deep learning learns a large number of labeled sample data through neural networks and extracts features; the resulting model is difficult to explain the correlation or causal relationship between its input and output in clinical practice due to a lack of process interpretability, and it is difficult to support medical diagnosis or causal reasoning in medical research [24–28].

Interpretability has become a difficult problem in the development and application of deep learning in the field of medical image processing. Therefore, in order to address the above issues, this article provides a detailed comparative analysis of state-of-the-art AI applications in medical imaging systems. The main contributions are as follows:

- Combined the development trend of deep learning in medical image processing, the application status, and problems faced by deep learning in the medical field are firstly reviewed.
- The connotation of deep learning interpretability is discussed, and the focus is on the research methods of deep learning interpretability.
- Advancement and the research progress of deep learning interpretability in medical image processing with particularity.
- Finally, the development trend of deep learning interpretability research in medical image processing is examined.

The remainder of this article is organized as follows: in Section 2, the interpretability problems and opportunities are discussed. In Section 3, the connotation of various imaging diagnosis methods is discussed. In Section 4, the methodology for interpretability in medical diagnosis is described and various methods are classified. In Section 5, the visualization model for a medical disease (e.g., glaucoma) is discussed in detail using deep learning, and CNN methods and procedures are explained in steps. In Section 6, the summary is discussed, while Section 7 concludes the paper.

## 2. Deep Learning Interpretability Problems and Opportunities

Many models of deep learning, such as the convolutional neural network (CNN), the deep belief network (DBN), etc., have been widely used in medical image processing. The researchers automatically extracted the feature information of Alzheimer's disease (AD) in brain images through deep learning methods, captured the brain changes caused by AD [4], and combined other multimodal information to diagnose mild cognitive impairment (AD/MCI) [5]. Lung cancer cells are automatically detected by deep learning [6], and the image blocks and pretrained CNN are combined to complete the classification of breast cancer tissue [7]. Through CNN, the low-level image data is transformed into a feature vector fused with nonimage modal data [8], and the nonlinear correlation between all modalities of the neural network is jointly learned to complete the diagnosis and prediction of cervical dysplasia. Automatic extraction of microaneurysm features [9], retinal blood vessel segmentation [10], and retinopathy classification [11]. These auxiliary diagnosis systems complete the rapid screening and diagnosis of diseases through deep learning, which greatly shortens the diagnosis time, reduces the diagnosis cost, and greatly improves the accuracy.

The medical image processing technology based on deep learning has made great progress, and at the same time, it has triggered people's thinking and research on the interpretability of deep learning. The author investigated the interpretability of deep learning published in machine learning and artificial intelligence (AI) related conferences (CVPR, ICML, NIPS, AAAI, ICCV, and IJCAI) and the top international medical imaging academic conference, MICCAI, from 2016 to 2020. Research papers on the interpretability and deep learning of medical image processing. Statistical analysis was done on related papers with the keywords explain, interpretable, and understanding in the title. The results are shown in Table 1.

After screening, a total of 212 related research papers were obtained. In general, deep learning interpretability is increasingly recognized as an important problem to be solved. Before 2015, there were almost no research papers related to deep learning interpretability. In 2016, there were only 11 related research papers, and in 2018, the number increased to 78. In 2019, deep learning interpretability is still a research hotspot. On MICCAI, the deep learning interpretability of medical image processing has also gradually attracted attention. In 2018, MICCAI accepted 3 papers related to deep learning interpretability, and in 2019, MICCAI set up a special working group to discuss the deep learning interpretability of medical image processing.

## 3. Connotation

At present, there is no unified definition of interpretability. In a broad sense, interpretability refers to obtaining enough information to be understood when one thing needs to be understood or solved. References [12, 29] define interpretability as the degree to which humans understand the reasons for decisions. The more interpretable the model is, the easier the decisions or predictions made are to be understood by humans. At the top international conferences on machine learning, some scholars have given various understandings of the interpretability of deep learning from the perspectives of methods and goals. For example, at the 31st Conference on Advances in Neural Information Processing Systems (NIPS) in 2017, RAHIMI, winner of the Test of Time Award, proposed that applying deep learning to certain fields raises questions about transparency and believes [29] that the key to AI is the ability and process to explain decisions, recommendations, predictions, or behaviors, and if the operation of the system is understood, then the system is interpretable. In addition, interpretability is a human-centered explanation process, and the ultimate goal is to make humans understand. Therefore, the connection mode, operation mode, and information processing mode of human brain neurons may affect the study of deep learning explainability.

**Figure 2:**
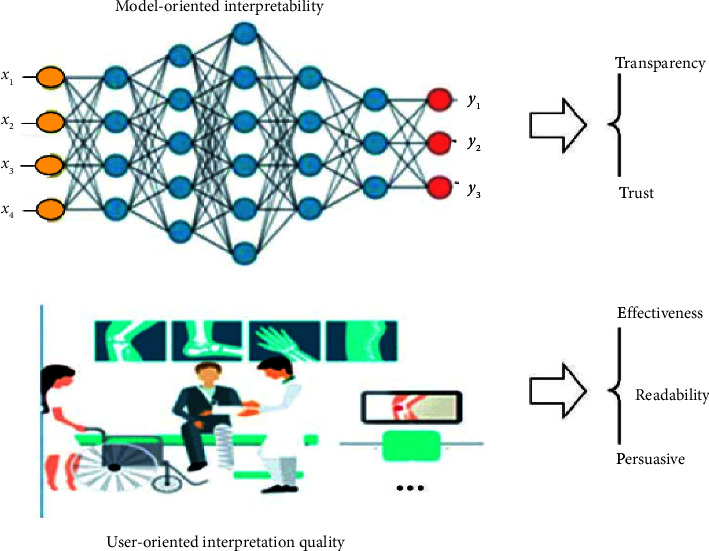
Objective illustrations of AI [13].

Traditional machine learning models based on statistical analysis have better interpretability. For example, traditional linear models can understand the meaning of parameters in neural networks and their importance and fluctuation range from the perspective of weights. User-friendly decision tree models will show its decision basis through a decision sequence. Variable screening criteria based on information theory help to understand which variables play a more significant role in the model decision-making process. Rule-based expert systems rely on domain-specific classification knowledge bases and a separate strategy library [29]. However, the structure of deep learning models is becoming more complex. For a multilayer neural network model that is superimposed by multiple nonlinear functions, it is also difficult to explain its decision-making basis, and it is difficult to directly understand the “brain circuit” of the neural network. Therefore, the goal of [13] is usually divided into two types: model-oriented and user-oriented (see Figure 2).

### 3.1. Model-Oriented Interpretability

When researchers debug machine learning models, they treat them as black boxes. Only seeing the input and output, it is difficult to understand the internal working principles of the black box, which makes it difficult to predict and debug the output results of the machine learning model, etc., which ultimately affects the in-depth understanding of the machine learning model and the further improvement of the results. Model interpretability focuses on transparency and trust.

### 3.2. User-Oriented Interpretation Quality

In many areas, interpretation is required when presenting the results to the average user. Ordinary recommender systems [14] provide item prediction and recommendation by collecting the information preferences of each user and using different information sources, usually only giving simple and intuitive reasons, which cannot be trusted by users. In order to make users better understand the prediction and recommendation results, some explainable recommender systems [15] include users in the interpretable category so that users can understand the reasons for making corresponding decisions, thus greatly improving the effectiveness of the recommendation results and enhancing the persuasiveness of decision-making. In the computer-aided diagnosis system, although the ability of the complex deep learning model to explain the decision is satisfactory [16], the quality of its interpretation and the readability and validity of the results are not high.

The objective laws that allow human beings to understand the world and explore things are mainly based on the thinking mode of causal inference. The rules in [17] can be generalized to complex environments. The practice has proved that the objective laws discovered based on causal inference in scientific exploration have strong generalization ability.

Based on the above understanding, the author attempts to generalize the interpretability of deep learning in a specific field as follows: people with knowledge in a specific field can grasp the degree of the causal relationship between the input and output of the deep learning model within the range of cognitive burden, including subjective, cognitive, and objective factors and their connotations, as shown in Table 2.

## 4. Methodology

The deep learning model consists of input, an intermediate hidden layer, and output. Each neuron in the intermediate hidden layer is composed of a linear combination of the previous layer and a nonlinear function. Although the values of the parameters and the training process are known. However, because the middle hidden layer is highly nonlinear, it is impossible to understand the specific meaning and behavior of the deep learning model. The purpose of deep learning is to discover knowledge and laws from sample data and solve practical problems, while the hierarchical combination of neurons in a neural network is to understand the operation mode of a neural network from the perspective of material composition. Understandable data information or model information helps find ways to understand and solve problems. The above can be summarized as interpretability research methods, and the mainstream directions of deep learning interpretability research methods are shown in Tables 3 and 4.

### 4.1. Visualization

Visualization is the display of data in large datasets in easy-to-understand ways such as graphics, images, and animations. It is one of the most intuitive ways to explore cognitive factors that can be explained by deep learning. By mapping abstract data into images and establishing a visual representation of the model, researchers can reduce the cognitive difficulty of deep learning models and understand the internal expressions of deep learning, thereby reducing the complexity of the model and improving transparency. Existing research mainly focuses on input data visualization and modeling internal visualization.

#### 4.1.1. Input Data Visualization

Deep learning can discover knowledge and rules from data and perform visual analysis of input sample data before modeling, which can quickly and comprehensively understand the distribution characteristics of data and facilitate understanding of problems. Reference [18] used the maximum mean discrepancy (MMD) method to find representative and nonrepresentative data samples to better understand the data distribution.

#### 4.1.2. Visualization inside the Model

The black-box nature of deep learning is mainly due to the high nonlinearity of the intermediate hidden layers. Existing research improves the transparency of the black box by visualizing internal neurons [19–21], filters [22, 23], and intermediate hidden layers [19, 24]. Reference [19] visualized the internal neurons of the deep neural network through two methods of activation maximization and sampling and tried to find the maximum input image of the activated filter, which can effectively display a specific pixel area and is interpretable. At the same time, through the inverse process of de-pooling, de-activation, and de-convolution to visualize the inside of the convolutional network, it is found that the low-level corresponds to corner or color features and texture features, and the high-level corresponds to local parts such as dog faces and wheels. The overall recognition ability is strong [20]. Images are learned through feature inversion, using the visual clarity of natural image priors to reconstruct the intermediate activations of the network. The visualization system in [21] can visualize neuron clusters by extracting the characteristics of neurons and connecting each neuron. Google Brain's feature visualization tool Lucid [23] can show the individual neurons within a deep learning network and their division of labor, helping to understand how neurons within a network are used as detectors for objects (such as buttons, clothes, and buildings), how they are stacked between network layers, and how they can become complex. These visualization methods can not only display the detection results but also visually observe the output contribution of each neuron in the neural network.

The intuitive expression of visualization reduces the complexity of the deep learning model to a certain extent and improves the transparency of the model, but it cannot be associated with higher-level semantics and requires high human cognitive ability, so there is still a certain degree of difficulty in interpretation.

### 4.2. Semantic Data

Semantics refers to the interpretation and logical representation of data. Semanticization refers to interpreting the semantics of hidden layer neurons in deep learning models through methods such as quantification or learning.

#### 4.2.1. Quantification of Neuron or Layer in Semantic Correlation

In order to understand the semantics learned by the network, reference [24] proposed a network dissection (ND) method by analyzing the correlation between the internal neurons or intermediate hidden layers of the neural network and human semantics and quantifying it. First, by collecting hierarchical semantic annotation data from different data sources, a dataset containing a large number of visual semantics is established. Then, the correlation between hidden layer units and semantics is quantified by using intersection over union (IoU), and finally, learn semantics about colors, textures, materials, parts, objects, scenes, etc. from the intermediate hidden layers. Reference [25] explored the semantics of the combined representation of multiple filters by studying the vector embedding relationship between semantics and corresponding filters [26] and maximizes the semantics encoded by the recognition filter by the concept activation vector. Reference [27] combined feature visualization and a semantic dictionary to study the decision-making network and the internal impact mechanism of the neural network on the output.

#### 4.2.2. Coding Learning Semantics

The internal neurons of the neural network diagnose and modify the neural network at the semantic level by learning semantics so that it matches the human knowledge framework and has a clear symbolic internal knowledge expression. Reference [28] created capsule networks whose internal neuron activity represents various attributes of specific entities appearing in images and trained them on the MNIST dataset, proving that capsule networks can encode some specific semantics, such as stroke scale, thickness, oblique angle, width, translation, etc. The information-maximizing generative adversarial network (InfoGAN) [36] divides the generator input variables of the network into incompressible noise and latent semantic code. The MNIST dataset [37] successfully encodes digit type, rotation, and width semantic information. The CelebA dataset [38] encodes the emotional part of the face dataset; the SVHN dataset [39] encodes lighting conditions and tablet environments; the 3D face dataset [40] encodes orientation, glasses, hairstyle, and mood; and the 3D chair dataset [41] encodes width and three-dimensional rotation information. The above datasets all learn semantics by encoding internal neurons, which makes it easier to understand the internal expressions of the model.

The deep learning model realizes end-to-end learning, which requires explaining the generation process of the deep learning model from low-level semantics to high-level semantics, which is not only conducive to understanding the specific structure of the neural network but also assists deep learning to make parameter adjustment truly controllable and feasible.

### 4.3. Quantification of Logical Relationships

Quantification of logical relationship is a judgment method to study the relationship between things. The relationships within or among things are related, juxtaposed, primary and secondary, progressive and causal, etc. The strength of the relationship can indicate the logical reasoning ability within or among things. For example, the causal relationship between input and output has strong reasoning abilities, which can show interpretability better than ordinary correlation. At present, there are three main types of research based on the logic relationship: end-end logic relationship, middle-end logic relationship, and the correlation of neurons within the model.

#### 4.3.1. End-to-End Logical Relationship

In order to find the pixels in the image that have the greatest impact on the deep learning results, the logical relationship between the input and the output is judged by studying the influence of the input layer changes on the output results. Using backpropagation [19] and combining gradients, network weights, or activations [42, 43] track information, and the network output tracks its input or intermediate hidden layers. Reference [43] filtered gradients through an optimization process to further extract fine-grained regions for specific prediction evidence. The core of these methods is to find the most representative perturbations through detailed search or optimization. In addition, the influence of occlusion on the output of each method is analyzed by inputting perturbed networks with regular or random occlusion [19, 33] and some samples [19, 30, 31, 44]. For example, reference [30] used meta-learning as an explanatory factor to establish perturbations to optimize the spatial perturbation mask and, through perturbation experiments, found features that had a greater impact on the output results and gradually established a linearly separable model [31]. Since it is impossible to see all the perturbations, it is necessary to find representative perturbations. Reference [32] used the statistical influence function to analyze the influence of increasing the weight of training samples or applying slight perturbations to the training samples on the loss function of a specific test sample in order to better understand the predictive performance of deep learning models.

The above methods all explain the results by exploring the mapping relationship between input and output. This sensitivity method of measuring the importance of variables/samples attributes interpretability to input features or samples, which is easier to understand but also tends to lead to different interpretable reasons for the same prediction results and is less stable. These methods are based on model agnosticism; they do not consider the internal structure of the model, do not open the black box, ignore the research on the structure of the middle hidden layer, and cannot understand the internal working mechanism of the model.

#### 4.3.2. End-to-End Logical Relationship

Studying the logical relationship between the intermediate hidden layer and the output of a deep learning model is a necessary process to further explore the internal working mechanisms of the model. Some studies use simpler, interpretable models to establish logical relationships with outputs by locally approximating the intermediate hidden layers of deep learning. For example, the gradient-based method and the local interpretable model-agnostic explanations (lime) method proposed by [33] use a linear model to establish a local mid-end logical relationship near the prediction result. Reference [34] used learning networks to perform deep neural network learning through regularized approximations of decision trees. Reference [35] proposed an interpretable CNN for end-to-end learning, adding a priori constraints with filters to achieve automatic regression to a specific object (such as a bird's head, beak, and legs) after training and separating them in the top layer of the convolutional layer. Then, the representation of the neural network is refined into a decision tree structure [45], each decision mode hidden in the fully connected layer of CNN is encoded from coarse to fine, and the decision tree is used to approximate the final decision result. Reference [46] used a finite-state machine (FSA) with interpretable sequence data to learn a recurrent neural network (RNN), taking the learning result as an interpretable structure. Reference [47] proposed a pooling operator commonly used in regions with CNN features (RCNN), which is replaced by an AND-OR graph (AOG) parsing operator. During detection, the bounding box is interpreted with the best parse tree obtained in real time from the AOG. In addition, there are studies in the reinforcement learning process that use the causal model [48] structure to encode the causal relationship of the variables of interest and use the causal model-based counterfactual analysis method to explain reinforcement learning.

Through the above interpretable methods, the internal mechanism of each deep learning model is approximated, the logical relationship between the local part and the output is established, and the objective interpretability is strong.

#### 4.3.3. Relationship between Neurons

Studying the relationship between internal neurons is of great significance for understanding the internal mechanisms of deep learning models. By identifying key data paths [49] and using piecewise linear functions [50] to analyze the functions of the corresponding layers of the model, the activation of neurons during training is detected and the relationships between different neurons are found. References [51, 52] transformed CNN into a graph model and explained the hierarchy and knowledge structure of CNN by automatically learning an explanation graph with tens of thousands of nodes. Each node in the explanation graph represents a partial pattern of an object in a convolutional layer in the CNN, and the knowledge graph is used to explain the decision. This type of approach explores the relationship between unknown neural network components by exploring the interrelationships of neurons within complex networks, understanding the training process and decision-making process within deep learning, but this relationship is only part of the underlying causal relationship, the topology of the neural network. The structure remains complex.

The deep learning model has a complex structure, huge parameters, and a heavy cognitive load. Visualization methods and semantic quantification methods cannot effectively explain the causal reasoning of the decisions made by the model. Therefore, analyzing objective factors with the method of causal reasoning is helpful to understand deep learning. The training and decision-making processes of the model realizes its internal transparency.

### 4.4. Interactive

Interaction refers to understanding the decision-making process within deep learning through the interaction of domain experts with the deep learning process of human-computer interaction through visualization tools [24, 33]. Humans are more sensitive to the interaction logic of objects and environments than to low-level semantic interactions such as color and texture. The deep learning system is modularized and customized with various advanced semantic deep learning modules [53, 54], and then these modules are combined according to cognitive logic to finally complete specific tasks. Reference [54] proposed deep intervention in the training and verification of the internal neural network. Based on GAN, the internal neurons of the neural network were modularized into natural images. When the model was diagnosed, the deep network was directly activated or combined with visualization tools. First, activate the neurons or neuron groups in the deep network, and through interactive, interpretable, experimental exploration, the internal modularization and customization of the deep learning model are realized to a certain extent.

## 5. Medical Image Processing Using Deep Learning

In the medical field, the examination and diagnosis of diseases mostly need to refer to medical images, which are highly dependent on imaging equipment and imaging environments. Compared with natural images, medical images are more complex, which is manifested in the following: (1) there are many types of images with large differences, and it is difficult to merge them; (2) most of the images are nonvisible light imaging (such as X-ray), which usually shows the intensity value of a special signal, the signal-to-noise ratio is low; (3) the color, grayscale, texture, and other appearance differences between the target and nontarget areas such as lesions are small; (4) the image pixels are large, and the target itself lacks fixed size, shape, grayscale, texture, and other apparent features, and there are great differences due to differences in individuals, imaging principles, and imaging environments; and (5) due to the influence of imaging principles and imaging environments, the images contain various artifacts.

At the same time, medical data is presented in multiple modalities, each with its own strengths and interrelatedness, such as between different diseases, between different diseases, between one disease and multiple diseases, between multiple diseases and the same disease, and so on, greatly limiting the prediction and diagnosis of the disease.

The introduction of deep learning into the medical field has greatly improved the feature extraction ability, screening level, and diagnostic efficiency of medical images. However, the data-driven, deep learning-assisted disease diagnosis and screening system can only output a single diagnosis or screening result, cannot provide a decision-making basis, is difficult to adopt, and is not friendly to algorithm personnel. Although deep learning interpretability research has achieved a large number of impressive results, most of them focus on specific models, and their interpretability also focuses on algorithm designers rather than doctors, medical researchers, and patients, which greatly limits medical diagnostic systems.

Deep learning interpretability research for medical image processing can provide an effective and interactive way for the deep integration of medical knowledge and disease-aided diagnosis with large-scale screening systems and effectively promote the intelligence of medical care. Different from the commonly used deep learning interpretability research methods, the deep learning interpretability research methods of medical image processing are not only affected by data but also related to the knowledge of doctors. Therefore, the two are similar and different in their research methods. The main differences are:

1. In terms of visualization methods, the interpretability of deep learning focuses on the visualization of sample data rules and the visualization of internal models. Medical imaging focuses on the lesion area, requiring intuitive reading.
2. In terms of semantic methods, the interpretability of deep learning focuses on the semantic information represented by the internal neurons or intermediate hidden layers of the model, while most medical images need to use natural language to simulate the doctor's decision-making process. At the same time, it is necessary to generate understandable decision-making processes and decision-making results, such as primary diagnosis reports.
3. In terms of logical relationship quantification, the interpretability of deep learning focuses on the logical relationship between input sample data and output results, between neurons within the model, and between neurons within the model and output results, while medical imaging is more Much attention has been paid to interpreting diagnoses with medical knowledge.

The following are some recent research trends of deep learning interpretability in medical image processing:

### 5.1. Visualization of the Lesion Area

**Figure 3:**
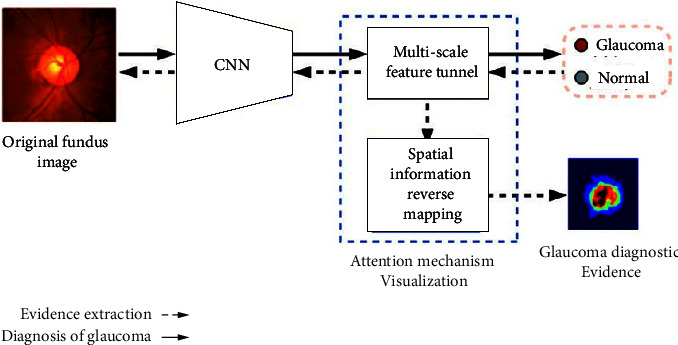
Visualization model [57].

**Figure 4:**
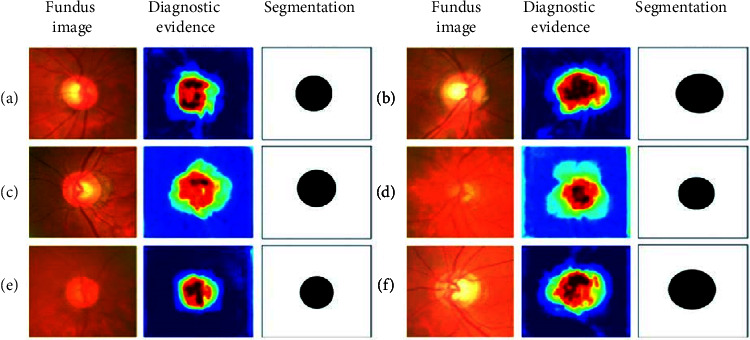
Expressions and illustrations of qualitative and quantitative [57].

The visualization of the lesion area mainly refers to finding out the lesion area and providing visual evidence through methods such as a heat map [55], attention mechanisms [56–58], and other methods [59, 60] so as to explore the medical science that provides the basis for decision-making. For example, reference [55] used the model to activate the fine-grained Logit heatmap to explain the medical imaging decision-making process. Reference [56] proposed an interpretable deep learning framework for detecting acute intracranial hemorrhage from head CT scan data by simulating the radiology workflow and iterating to generate attention maps, using class activation maps [42] from training retrieve forecast basis from data. Reference [57] weakly supervised the diagnosis of glaucoma based on the attention mechanism (see Figure 3), which provides a visual interpretation basis for the automatic detection of glaucoma (see Figure 4). In the process of automatic detection of glaucoma, the system gives three types of outputs: prediction result, attention map, and prediction basis, which enhance the interpretability of the results. When reference [58] detected early-stage squamous cell tumors, they focused on the interpretability of the results with the embedded activation map representation and used it as a constraint and provided a more detailed attention map through visualization methods. During basal cell carcinogenesis detection, an interpretation layer was designed as a digital staining method to bring together [59]. Reference [60] quantified the specificity of learned pathology through visualization methods on raw images, using task-specific interpretable features to differentiate clinical conditions and make the decision-making process transparent.

By using visualization methods to locate or quantify regions in real images, to provide visual evidence, to improve the perception of the internal representation capabilities of deep learning models, and to understand the model's decision-making basis.

### 5.2. Semantic Medical Records

**Figure 5:**
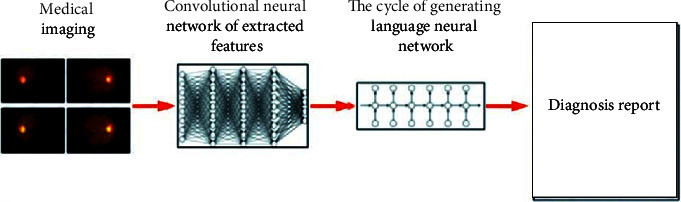
Mapping of medical images into reports [61–66].

At present, there are few research studies that introduce medical knowledge into the model and associate it with neurons. Most of them use natural language processing methods to integrate medical record information [61–66] into the image processing process. The image is directly mapped into a diagnostic report, giving an understandable diagnostic basis (see Figure 5).

Reference [61] proposed a multimodal medical imaging diagnostic model, which unified the imaging model and language model in the deep learning framework and established a mapping relationship between the two modalities of medical imaging and diagnostic report. In this way, the deep learning model can not only give the diagnosis result but also simulate the doctor's diagnosis and write the diagnosis report, so as to provide a comprehensible diagnosis basis. Based on the same method, reference [62] noted that radiologists would observe the symptoms of different diseases when interpreting images, such as liver metastases that spread to regional lymph nodes or other parts of the body, so they included associations with other diseases in the diagnosis report. Based on this, prior domain knowledge is first obtained from the text and then correlated with these symptoms to develop a multiobjective CAD framework for the detection of multiple diseases, which not only improves the performance of deep learning models but also provides a more accurate diagnostic report. In predicting high malignancy, reference [63] explained the semantic features of low-level radiologist models formed in an expert knowledge-driven manner by quantifying diagnostic features. Reference [64] utilized a GAN (consisting of an interpretable diagnostic network and a synthetic lesion generation network) to learn the relationship between tumors and standardized descriptions to accomplish an interpretable computer-aided diagnosis of breast masses. The MDNet model proposed by [65] integrates a variety of networks, designs a medical image diagnosis network based on semantic and visual interpretability, generates image representation, uses a long short-term memory network (LSTM) to extract semantic information, and generates more detailed verbatim images of areas of interest, but with high model complexity. Reference [66] further improved the model by inserting interpretable representations between two different neural networks and combining the two, first using a segmentation network to identify lesions from frequency-domain optical coherence tomography (OCT) images and then outputting the segmentation feature map. Then, take the segmentation feature map as input, use the tissue map with the diagnosis and the best referral to train the classification network, perform the classification, and output the diagnosis probability and referral recommendation. The experimental results and the expert clinical diagnosis results are important milestones in medical image interpretability research.

In the auxiliary diagnosis and screening of diseases, the deep integration of different deep learning models and medical knowledge can not only output the diagnosis results but also provide the basis for diagnosis decision-making for verification and comparison. If the diagnosis decision is inconsistent with deep learning or with the medical knowledge on which it is based, a better decision can be made through further analysis. If the doctor's decision is better, the deep learning model can be adjusted. Well, it enriches the doctor's knowledge and enables him to make better decisions.

### 5.3. Casual Inference Etiology

The logical relationship of deep learning interpretability lies in the causal inference of the data for the model designer, but no one knows what factors are based on the auxiliary diagnosis results.

**Figure 6:**
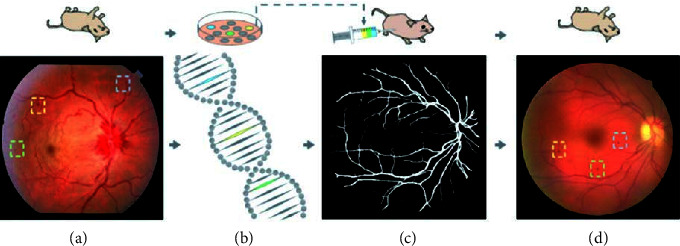
Illustrations of Koch's law [67].

References [67–69] explored the interpretability of convolutional neural networks in medical imaging by referring to Koch's law in the principles of infectious diseases. Koch's postulates (see Figure 6) state that by associating a certain lesion with a specific pathogen, the identification of infectious diseases is the gold standard for the identification of infectious disease etiology.

In addition, some scholars have introduced methods from other fields into the study of the interpretability of medical images. For example, reference [70] explained how to view individual features through a shared variable engine (SVE) in the detection of functional magnetic resonance imaging (fMRI) to identify autism spectrum disorders, combining image structure and shapely values in game theory. Reference [71] used deep probabilistic models to capture complex disease progression while leveraging attention mechanisms to improve clinical interpretability. Reference [72] proposed to explain the internal state of the neural network based on semantics and use the directional derivative quantization model to predict the underlying high-level semantics learned by the activation vector. Predicting the grade of diabetic retinopathy (DR) by fundus imaging and testing the importance of treatment methods such as microaneurysm (MA) and panretinal photocoagulation (PRP) in different DR grades.

Most of the above methods establish the interpretable basis of the model by introducing other fields to judge the causal relationship, which has a certain degree of interpretability, but the integration with medical knowledge is not enough. The method of causal judgement based on medical knowledge needs to be further explored.

## 6. Challenges and Future Directions

At this stage, the performance of deep learning models has greatly improved, but the complexity of the models has increased almost simultaneously, and interpretability has become a major problem in AI development. Although the interpretability research on deep learning has made some progress, it still needs further exploration, especially the interpretability research on deep learning in medical imaging, which is still in its infancy. Therefore, based on the analysis and understanding of current research practices, the author believes that the interpretability research of deep learning in medical imaging can be carried out from the following aspects in the future, and the explainable AI (XAI) methods can mitigate the risks by enhancing the diagnosis transparency and decision-making process [73].

### 6.1. Visualization of Lesion Characteristics

To study the transparency of deep learning, the current methods of visualizing input data, visualizing intermediate hidden layers, and visualizing feature maps of high convolutional layers have increased the transparency of deep learning models to a certain extent. By improving the visualization inside the deep learning model and integrating the visual feature map with medical knowledge, the basis of the model's decision-making is deeply excavated to improve the deep learning interpretability of medical image processing, which reduces the cognitive difficulty of the model. It is very important to improve cognitive ability.

### 6.2. Semantic Medical Images

Most existing semantically interpretable methods combine image recognition with natural language processing to generate understandable diagnostic reports. Natural language processing uses deep learning methods, which are equivalent to explaining black boxes with black boxes. Although semantic information can be obtained, the model is agnostic. The current development in transfer learning, semantic segmentation, and other directions has greatly promoted the interpretable research of deep learning. At the same time, combining the semantic method inside the model with multi-modal medical data may be another way of doing semantic medical imaging [74].

### 6.3. Causal Reasoning on Medical Rules

On the basis of logical reasoning, the knowledge graph, as a highly readable external knowledge carrier, provides a great possibility to improve the interpretability of algorithms. Using an imaging neural network to build a medical diagnosis knowledge map, combined with the image feature extraction ability of a deep convolutional neural network, improves the model's domain knowledge matching ability and knowledge logical reasoning ability, making it possible to advance AI medical diagnosis from intuitive learning to logical learning.

### 6.4. Interactive Research

How to establish interaction between domain experts, model designers, and deep learning models is critical to improving interpretability. In-depth intervention in the design of the internal training phase and verification phase of the neural network through the modularization of the internal neurons of the neural network and the use of visual tools to interactively explore the various stages of deep learning, find the impact of interactive operations on model diagnosis, and realize the internal deep learning model. Deep feature extraction through modularization, if the high-level semantic definition can be successfully completed, especially by doctors, will enrich the objectivity of causal logic on the basis of being close to the cognitive level, thereby greatly improving the interpretability of deep learning.

## 7. Conclusion

The super performance of deep learning has promoted the huge development of AI applications. AI models can help doctors shorten the time it takes to read images and speed up diagnosis. However, the interpretability of algorithm conclusions is becoming more and more important, and understanding the algorithm decision-making process is helpful to build maximum understanding and trust between humans and machines. In recent years, the issue of interpretability has received wide attention from the government, industry, and academia. The U.S. Defense Advanced Research Projects Agency (DARPA) has funded the explainable AI project (XAI). It can be expected that when AI is interpretable, its efficient diagnosis speed and high accuracy level can free medical practitioners from repetitive and complicated diagnosis and treatment tasks. The intelligent diagnosis system provides a fast diagnosis for patients while providing an explainable diagnostic basis.

Based on the definition of interpretability, this paper introduces and analyzes the research status and progress of medical imaging deep learning interpretability, focusing on the existing deep learning interpretability research methods and deep learning interpretability research methods for medical image processing. It also briefly discusses the development direction of deep learning interpretability research in medical image processing, hoping to provide some help to researchers in related fields.

## Figures and Tables

**Table 1:** Summary of yearly literature.

Conference	2016	2017	2018	2019	2020	Total
CVPR	1	4	11	8	—	24
ICML	0	2	13	22	—	37
NIPS	8	12	22	8	—	50
AAAI	0	2	8	12	16	38
ICCV	2	3	9	8	—	22
IJCAI	0	8	12	21	—	41
MICCAI	0	0	3	7	—	10
Total	11	31	78	86	16	212

**Table 2:** Factors and connotations.

Factor	Connotation	Explanation
Subjective	Different domain knowledge, different cognition ability of causal relationship	Doctor: in the process of diagnosis, according to the medical knowledge he has mastered and combined with the clinical symptoms of the patient, he will comprehensively reason about the disease and explain the cause and pathology in detail
		Model designers: have deep learning model design and other related algorithm knowledge, and use deep learning directly as a black box. Based on statistics, explain the parameter tuning process and output results

Cognitive	Cognitive ability is determined by perception, memory ability, etc., which puts forward requirements for the complexity and scale of the model and the expression method of the model	Linear models can understand the importance of features from the perspective of weights, but how can it be understood by a series of linear and nonlinear combinations of neural networks
		A simple decision tree model can quickly and intuitively get interpretable results, but a complex decision tree puts forward higher requirements on human perception and memory

Objective	Accurately judging and refining the causal relationship between the input and output of a deep learning model is an objective criterion for judging model interpretability	In the medical field, the diagnosis results are based on the current physical condition of the patient, the lesion characteristics of the medical imaging response, the clinical observation data and the medical knowledge of the doctor
		In the field of deep learning, how is the relationship between input samples and output results, how to quantify, how strong the relationship is, and how to approximate the causal relationship

**Table 3:** Evaluation of various interpretability methods.

Type	Illustrate	Categorize
Visualization	Input data	Subjective factor
	Model internal	Cognitive factor

Semantic	Quantitative	Subjective
	Encoding	Cognitive
Quantification of logical relationships	Middle-end logical relationship correlation	Subjective
Interactive	Control generative semantic	Subjective

**Table 4:** Summary of various state-of-the-art methods in medical imaging.

Reference	Mathematical models and methods	Applicable scene	Data set	Result	Limitations and basis
[30]	Image enhancement by wavelet transform and improved AlexNet network structure for ultrasound image segmentation	Wavelet transform, which has an excellent effect in image enhancement; AlexNet has low network complexity and fewer parameters, and performs well in the case of insufficient samples	325 breast ultrasound images provided by the ultrasound department of a tertiary hospital	The indicators of the segmentation results are better than the existing methods	The model only uses 325 original images, and the sample data set is too small, resulting in poor robustness and adaptability; the extracted abstract features are limited, which limits the segmentation ability of the full convolutional network

[31]	Improving convolutional neural networks using residual learning, sobel enhancement + GLCM	It can effectively solve the problem of network degradation and gradient fragmentation	Intravascular ultrasound images of 63 patients with carotid atherosclerosis marked by professional physicians in the fourth military medical university	87.1% accuracy	Using the residual network and increasing the depth of the network, the neural network model becomes more complex

[32]	Improved CNN architecture	Video image classification	Video image of an echocardiogram	A classification accuracy of 92.10% can be achieved	The generalization ability is weak, two 2D CNNs are used, and the interframe motion information of the time dimension is not considered

[33]	Deep DenseNet and transfer learning	It can alleviate the problem of gradient disappearance and strengthen feature propagation; efficiently reuse features; and reduce the number of parameters	7230 ultrasonic images of 6 abdominal organs	Classification accuracy rate 86.40%	Network training takes a long time, and DenseNet uses dense connections, which increases the amount of network parameters and calculations

[34]	Improved U-Net's modified video segmentation algorithm	Suitable for image segmentation, especially in medical image segmentation	7230 ultrasonic images of 6 abdominal organs	Segmentation accuracy rate 81.72%	The guidance algorithm for identifying and locating the liver is not reasonable and effective, and the guidance method that only increases the liver area by moving the probe

[35]	Automatic detection of intima and media-adventitia boundaries in coronary IVUS images based on deep convolutional networks DFCN-1 and DFCN-2, combined with stacked funnel networks and generative adversarial learning for automatic detection of key tissue boundaries	Stacked funnel network can achieve automatic labeling, and generative adversarial learning can alleviate the problem that deep convolutional networks require a large number of labeled medical ultrasound images	435 20 MHz IVUS images of the international standard IVUS image public database SetB	The test result is 94.00%	Insufficient anti-interference ability and cross-domain segmentation generalization ability; low-lying, high-lying and other pixel-level regional errors are difficult to avoid

## Data Availability

The data supporting the findings of this study are available within this article.
